# Multidisciplinary Case Management in Mesiodens Impacted Cases with Calcifying Odontogenic Cyst

**DOI:** 10.1155/2022/4084857

**Published:** 2022-08-04

**Authors:** Tania Saskianti, Udijanto Tedjosaongko, Regina Ayu Pramudita, Nita Naomi, Alit Rahma Restu

**Affiliations:** Department of Pediatric Dentistry, Faculty of Dentistry, Airlangga University, Surabaya 60132, Indonesia

## Abstract

Calcifying odontogenic cyst (COC) is a cyst originating from odontogenic epithelium and has a characteristic ghost cell appearance. Clinically, COC is characterized by asymptomatic swelling, leading to lingual expansion, tooth migration, apical resorption, and perforation of cortical bone. COC is most often asymptomatic and is often only discovered during a routine radiological examination. This case report describes the clinical interventions to manage a patient with mesiodens impacted with COC. The procedure is aimed at evaluating the multidisciplinary management of COC at Universitas Airlangga Hospital, Surabaya. Multidisciplinary care has an important role in treating COC cases comprehensively.

## 1. Introduction

The calcifying odontogenic cyst is an uncommon benign cyst of odontogenic origin, characterized by an ameloblastoma-like epithelium with ghost cells that may calcify, first described by Gorlin et al. in 1962 [1].

Clinically, it is characterized by slowly growing asymptomatic swelling. It may have a central (intraosseous), or less frequently, a peripheral (extraosseous) localization. It may cause lingual expansion, displacement of teeth, root resorption, and perforation in the cortical bone [2].

On radiographs, COC appears as a well-limited unilocular radiolucent lesion of different sizes, shapes, and opacity levels. Impacted teeth may be associated. Root resorption, root divergence, or cortical blowing may be observed [3].

A definitive diagnosis of calcifying odontogenic cyst can be reliably made based on a histological examination. An abnormal form of keratinization in the form of the ghost cells is the most distinguishing feature of a COC. However, their presence does not confirm the diagnosis as other lesions show similar presentation. Hence, a diagnosis of COC should only be made for a lesion in which the formation of ghost cells takes place in a typical epithelial cyst lining, presenting a basal layer of cuboidal or short cylindrical cells and an overlying layer consisting of cells that bear resemblance to stellate reticulum-like cells [4].

The recommended treatment for COC consists of enucleation with curettage, which means enucleation followed by removal of a 1 to 2 mm layer of bone around the periphery of the cystic cavity with a sharp curette or a bone bur. This procedure is aimed at limiting the risk of recurrence. Decompression and marsupialization are conservative treatments used in large lesions with a high success rate [5].

In this case, the cyst has involved the apical part of the surrounding teeth, so multidisciplinary treatment is needed for comprehensive treatment to remove the cyst and preserve the tooth. The purpose of this report is to present an interesting and rare case of COC associated with impacted mesiodens and to highlight the importance of multidisciplinary management of COC.

## 2. Case Report

A 14-year-old boy came to the Pediatric Dental Clinic of Rumah Sakit Gigi dan Mulut Universitas Airlangga with a history of fractured teeth due to an accident 5 years ago. The patient is currently on apexification treatment.

**Figure 1:**
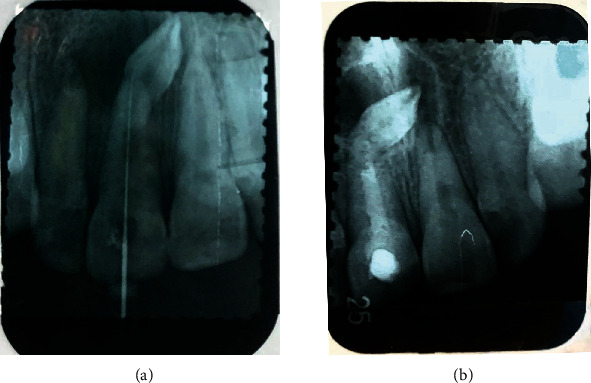
Periapical radiograph: (a) #11 with open apex; (b) one year later found apexification material on root canal and closed apex. The impacted mesiodens shown surrounded with cyst between apical of #21 and #11.

**Figure 2:**
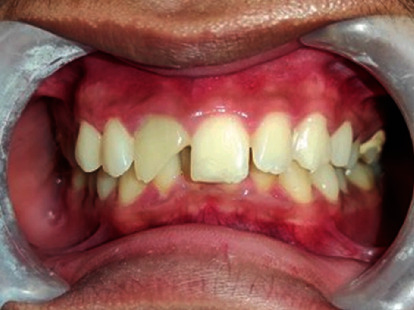
Clinical features. Intraoral photography highlighting #11 with class III Ellis crown fracture classification.

Periapical radiographic examination showed #11 with an open apex (Figure 1(a)), and the patient was scheduled for obturation. During treatment, the patient felt recurrent pain on #11. On clinical examination, #11 was found with an intact temporary restoration, negative percussion, and palpation test. Based on clinical examination, the provisional diagnosis is pulp necrosis with class III Ellis crown fracture classification (Figure 2). Radiograph examination revealed #11 with a closed apex and well-defined margin radiolucency area around mesiodens between apicals of #21 and #11 (Figure 1(b)).

**Figure 3:**
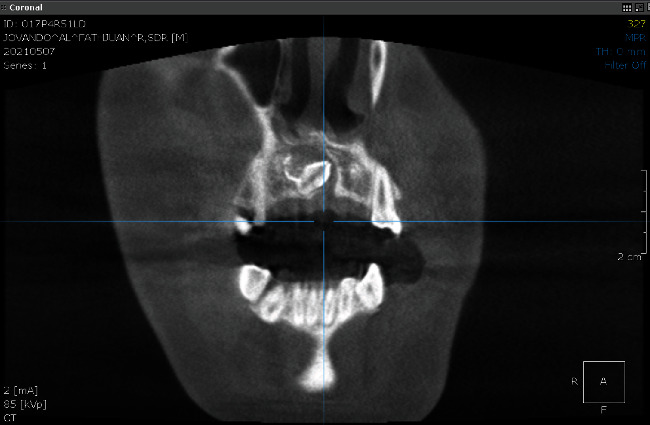
CBCT radiographic view. The mesiodens impacted tooth was seen between teeth 11 and 21 in an inverted position. Mesiodens tooth distance with the base of the nasal cavity: 3.93 mm. A mixed radiolucent-radiopaque image is seen around the crown of the mesiodens with a clear border with radiopaque edges and an oval shape. The lesion appears to cause a discontinuity of the palatal cortical bone, and the lesion is in contact with the floor of the nasal cavity.

Interpretation of CBCT radiographic examination showed inverted mesiodens impaction on the palate of #11 and #21; the lesion was demarcated, radiopaque, oval in shape, and causing a discontinuity in the base of the nasal cavity (Figure 3).

**Figure 4:**
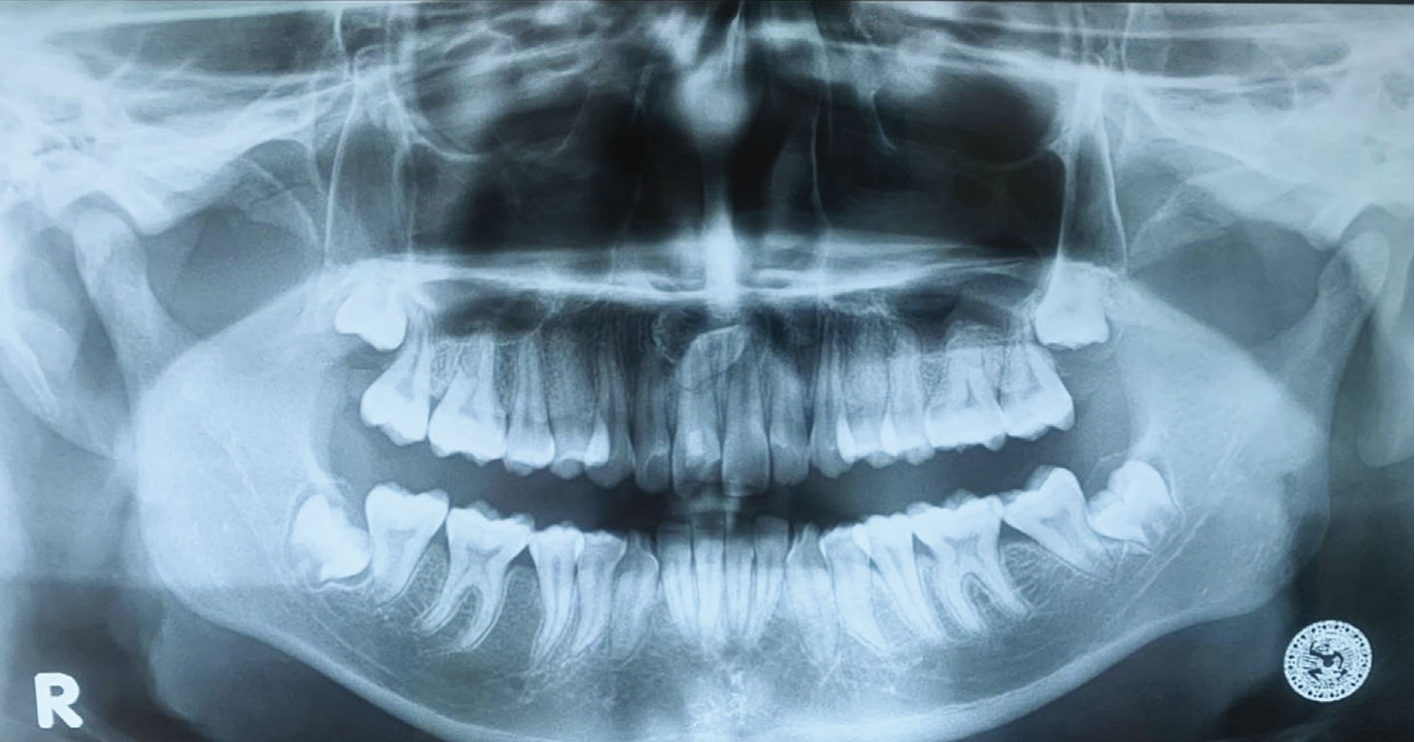
Panoramic radiographic view. Panoramic radiograph revealing a well-defined unilocular radiolucency around impacted mesiodens.

Based on periapical, panoramic, and CBCT, radiographs displayed unilocular, radiolucent, and well-delineated defect related to impacted mesiodens, suggesting a dentigerous cyst (Figure 4). The differential diagnoses are radicular cyst and nasopalatine duct cyst.

## 3. Case Management

Treatment was planned with a multidisciplinary approach with the oral and maxillofacial surgeon under general anesthesia for odontectomy of mesiodens impacted teeth and enucleation of the cyst, followed by apical resection of teeth involved teeth and closed with MTA plug.

**Figure 5:**
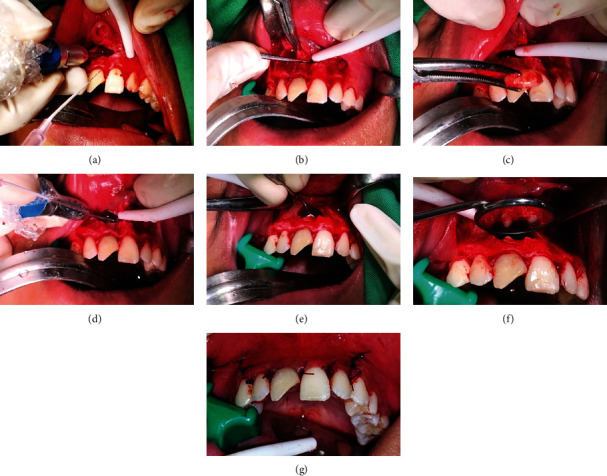
Surgical procedure: (a) buccal window; (b) curettes of granulation tissue; (c) extraction of mesiodens; (d) apex resection; (e) MTA and GIC application; (f) evaluation; (g) suturing.

Laboratory assessment and thorax radiographic examination were done before the procedure and found no negative symptoms. RT-PCR testing for SARS-CoV-2 detection from nasopharyngeal and oropharyngeal swabs to screen for both symptomatic and asymptomatic COVID-19 was done, and the result was negative. This test was mandatory for the preoperative procedure in the hospital. After confirming that the patient is completely anesthetized, an extraoral asepsis procedure was performed using 70% alcohol and 10% povidone-iodine intraorally. A vasoconstrictor consisting of 2 ampoules of pehacain diluted with 6 ml of saline, injected with a local infiltration technique using a 21 gauge syringe, was administered, and a full-thickness trapezium flap was elevated on a periapical area of #12, #11, #21, and #22 regions. A round carbide bur under constant irrigation for cooling was used to enlarge the bony defect to the buccal window to gain access to the periapical lesion and root end of the affected tooth (Figure 5(a)). Needle aspiration test showed mixed blood and cyst fluid; curettes were then used to remove the soft granulation tissue (Figure 5(b)), which further aided inadequate visualization of mesiodens to ensure complete extraction and no remaining dental follicle. Apex resection was initially planned for #11. However, the wall of the cyst was found extended to the adjacent teeth; hence, the apex resection was also performed on #21. The apical end of 2 mm was resected in faciolingual direction to the long axis of the tooth with a tapered fissured bur in a low-speed handpiece under constant irrigation (Figure 5(d)). The resected root surface was inspected, prepared, and then filled with mineral trioxide aggregate and glass ionomer cement on the periapical area (Figure 5(e)). Spongostan was then applied to the defect area, then sutured with silk Vicryl 4.0 (Figure 5(g)).

Surgical procedures also included germinectomy of 18, 28, 38, and 48 tooth buds.

**Figure 6:**
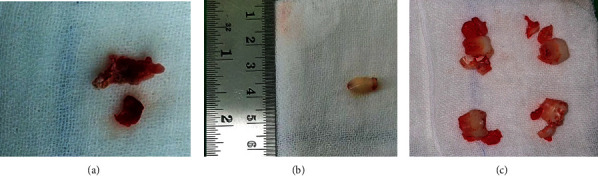
Specimen postoperative: (a) the cystic specimen after enucleation; (b) mesiodens; (c) tooth bud post germinectomy.

Histopathological anatomy results obtained after cyst enucleation and mesiodens collection (Figure 6) confirmed the diagnosis in the form of COC.

**Figure 7:**
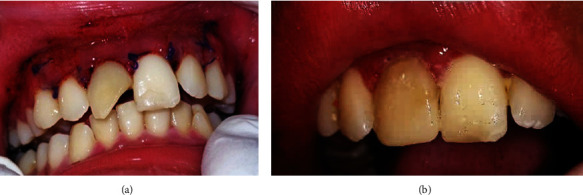
Postoperative intraoral view: (a) one day postoperative; (b) composite restoration on #21 and #11.

The patient was recalled after one day to access the surgical site recovery and found no sign of pain, dizziness, nausea, and vomiting. On extraoral examination, there were no signs of edema, hyperemia, and tenderness. On intraoral examination, the postoperative wounds on #12, #11, #21, #22, #18, #28, #38, and #48 were well sewn and hyperaemic, and minimal debris was seen at the edges of the sutures (Figure 7).

On palpation examination, the postoperative wound was palpated in the regions of #12, #11, #21, #22, #18, #28, #38, and #48, and there was minimal edema and tenderness at the edges of the sutures. On day 7 postoperative, the suture was removed. In #11, root canal access was completed without anesthesia and #21 with anesthesia. #11 and #21 were prepared up to sizes 40 and 50, respectively, 3 mm short of the apical foramen. In all teeth, the root canals were irrigated with 3 mL of 2.5% sodium hypochlorite (NaOCl), and distilled water at each change of file was used as an intracanal dressing. The quality of canal filling with the medication was confirmed radiographically. The teeth were temporarily restored with an intermediate restorative material.

After two times of sterilization, the intracanal dressing was removed, and the root canals of #11 and #21 were filled with gutta-percha and followed by composite resin restoration. Follow-up is scheduled at 3 months, 6 months, and 1 year to assess clinical and radiographic signs of healing.

**Figure 8:**
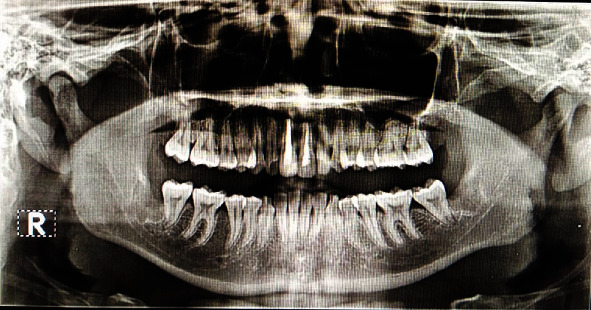
Radiographic examination 3 months postoperative.

**Figure 9:**
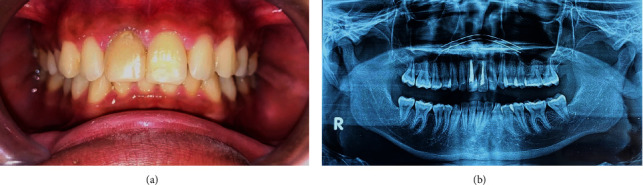
6 months postoperative recall: (a) intraoral view; (b) radiographic examination.

The patient came after 3 and 6 months postoperative; there were no signs of swelling, redness, or tooth mobility of #11 and #21 and underwent an X-ray panoramic control which described a radiopaque area with several radiolucent areas apical to #11; #12 with an impression of the bone ossification process showed a beginning bone regeneration. There are no signs of recurrence (Figures 8 and 9).

## 4. Discussion

The calcifying odontogenic cyst is an uncommon benign cyst of odontogenic origin [6]. Retrospective studies, case reports, or case series with a significant number of COC cases have been published [7–13].

The last 20 years from the following databases: PubMed, Medline Ovid, Web of Science, Scopus. The present systematic review revealed that COC patients' age ranged from two to 92 years, and the mean age at diagnosis was 30.7 (±21.0) years. Males made up 184 (51.8%) of the COC cases, while females made up 171 (48.2%) of the cases (male to female ratio: 1.0 : 1). Regarding anatomical location, a predilection for the maxilla (53.3%) was observed. The mandible was affected in 45.7% of cases, and information about the affected site was not available in only one case. COC was asymptomatic in 89.2% of patients. Symptom duration ranged from one to 180 months, with a mean of 18.1 (±29.8) months. Most cases exhibited a well-defined border (87.0%), with mixed (61.1%) and unilocular appearance (81.9%). The bone expansion was observed in 81.9% of cases. Lesion size ranged from 2.0 to 90.0 mm, with a mean of 29.5 (±20.8) mm. Regarding treatment, enucleation was the most common (69.9% of cases). In 194 cases (96.0%), no recurrence was reported. As regards the follow-up period, the mean duration of surveillance ranged from three to 264 months, with a mean of 42.2 (±43.6) months. Concerning treatment, enucleation was the modality of choice in most cases (85 cases, 77.1%) [14].

As regards treatment, conservative surgery was the main choice for COC. Herein, the two strategies most frequently reported for COC treatment were enucleation in 69.9% of cases and surgical removal or excisional biopsy in 18.5%. Furthermore, a two-stage approach was followed in 4.0% of cases. Uncommonly reported in COC therapy, this option is mainly chosen for other odontogenic cysts such as dentigerous or odontogenic keratocyst. Decompression or marsupialization is first performed to reduce intraluminal pressure and shrinkage of the cyst following a period of months. This allows bone growth and removal of the cyst in a second stage, with less risk of damage to important anatomical structures [15, 16].

The present review identified two COCs treated only by marsupialization. The recurrence rate after conservative COC management was low for both intraosseous and extraosseous lesions [14].

Considering the complexity of the case, we decided to manage the mesiodens impacted case with COC multidisciplinary [5]. Radiographically, COCs are well-circumscribed and present as a unilocular radiolucency with calcifications of varying density reported in one-third of cases [17].

In this case, radiographic examination revealed a solitary well-defined unilocular radiolucency around the impacted mesiodens. Based on the clinic-radiological findings, differential diagnosis of benign odontogenic lesions like a dentigerous cyst, radicular cyst, and nasopalatine duct cyst was considered. In the current case, with the presence of pericoronal radiolucency associated with unerupted mesiodens, the working diagnosis of the dentigerous cyst was made.

A definite diagnosis of calcifying odontogenic cyst can be reliably made based on a histological examination due to the lesion's lack of characteristic clinical and radiological features, as well as its variable biological behavior [19].

Histological features of the lesion show a fibrous capsule with a lining of odontogenic epithelium. The basal layer is made up of ameloblast-like columnar or cuboidal cells of 4–10 cell thickness. It is covered by loosely arranged epithelial cells bearing similarities to the stellate reticulum of the enamel organ. There are varying numbers of epithelial cells that are devoid of nuclei and eosinophilic and retain their basic cell outline (ghost cells). These ghost cells can calcify, and calcifications are constant but vary in number [6].

**Figure 10:**
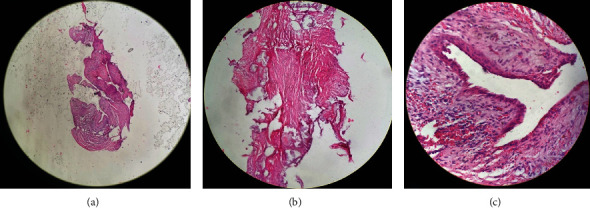
Histopathological examination: (a) showing cyst wall; (b) presence of ghost cells and calcification masses within the epithelium; (c) cuboidal and squamous epithelial cells.

The results of the histopathological examination on this case show sections of cyst wall tissue lined with thin squamous cuboidal epithelium, consisting of round, mononucleated, and smooth chromatin cells. The stroma is a fibrous connective tissue with many foci of eosinophilic calcification resembling ghost cells. There were no signs of malignancy (Figure 10).

The patient came with a history of trauma and swelling. Traumatic dental injuries to the teeth and the maxillofacial structures are a common occurrence, with the majority affecting the dentoalveolar structures. The most common types of traumatic dental injuries are crown fractures in the permanent dentition. An accurate diagnosis is essential to facilitate prompt management, which is widely regarded to have a major influence on the prognosis of the outcome.

Radiographic examination is essential for the diagnosis of traumatic dental injuries. In addition to a thorough clinical examination, intraoral radiographs such as periapical radiographs and upper standard occlusal radiographs are routinely prescribed to identify the location and nature of traumatic dental injuries. The diagnostic radiographs also form part of the baseline records to allow objective assessment at the follow-up appointment [20].

The clinical parameters observed were the presence of symptoms in the traumatized tooth, coronary discoloration, degree of tooth mobility, and the health of surrounding soft tissues, through visual examination and palpation. The radiographic parameters were the presence or absence of periapical lesion, pulp canal obliteration, and pathological root resorption, which was differentiated from physiological resorption by clinical history, patient age, and morphological characteristics of resorption. With both clinical and radiographic parameters, an analysis of both was performed to establish the condition of vitality or pulp necrosis [21].

Moreover, it has also been suggested that trauma diagnostics during childhood is more difficult than in grown-up persons. These authors reported that the sutures of the skull are wider and that more adipose tissue covers the bones, which will complicate the diagnostics accuracy. Fortunately, intraoral radiographs give a very low radiation dose if the correct technique and good X-ray equipment are used making it ethically indicated to prescribe intraoral radiographs for adults and children whenever the clinical examination indicates that a more severe dental injury might exist [22].

Recently, a new tomographic imaging method has started to be used, cone-beam CT (CBCT). CBCT has high diagnostic accuracy in cases of traumatic dental and maxillofacial injuries. But intraoral radiographs are a good start and are accurate enough if we only suspect dental injuries or minimally displaced bone fractures according to other researchers. In general, in dental trauma, CBCT should only be prescribed in selected cases, where conventional radiographs provide inadequate information for treatment planning [22].

In this case, after the periapical radiographic examination revealed mesiodens impaction on the apical of the maxillary anterior teeth, we referred the patient for a CBCT radiographic examination to help determine the diagnosis and location of the impacted tooth and appropriate treatment plan.

According to the periapical radiography, apical #11 was exposed with an open apical; then, apexification was performed by the pediatric dentist. Apexification is a method of treatment for immature permanent teeth in which root growth and development ceased due to pulp necrosis. Its purpose is to induce root-end closure with no canal wall thickening or continuous root lengthening. It can be achieved in two ways: (1) as a long-term procedure using calcium hydroxide dressing to allow the formation of a biologic hard tissue barrier or (2) as a short-term (more recent) procedure, creating an artificial apical plug of MTA or other bioceramic material [23].

The mechanism of action of calcium hydroxide in the induction of an apical barrier is still controversial, although it is formed by cells originating from the adjacent connective tissue. The calcified barrier, even when appearing radiographically and clinically complete, is histologically porous and may be composed of cementum, dentin, bone, or osteodentin. This procedure requires multiple visits and could take a year or more to achieve a complete apical barrier that would allow root canal filling using gutta-percha (GP) and sealer [24].

**Figure 11:**
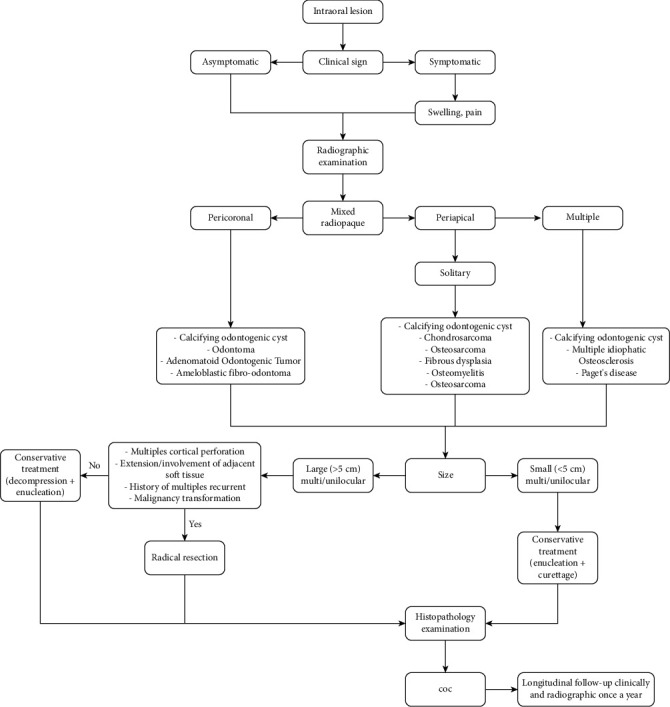
Algorithm to diagnose and manage cystic lesions [18].

Cyst size is an important factor when formulating a treatment plan. The cystic lesions having a size < 5 cm can be managed with simple enucleation/curettage and submitted for pathologic examination (excisional biopsy) while preserving the strategic tooth or teeth involved. In the few cases that had clinical or radiographic evidence of multilocular lesions, extensive lesions with involvement of adjacent soft tissues or a history of multiple recurrent lesions should be considered to have aggressive behavior, and radical resection could be the first choice of treatment option depending on the surgeon's training, available resources, and the patient's preferences. Otherwise, conservative treatment methods such as decompression with a second surgical procedure (enucleation) and another aggressive approach than bone resection can be chosen, and the failures of this treatment must indicate the radical resection method as well as for extensive lesions without aggressive behaviors. Longitudinal follow-up should be considered [18, 25] (Figure 11).

The recommended treatment for COC, in general, is total excision in one step. Enucleation associated with curettage is the usual therapy when referring to the cystic variant. Hence, after removing the lesion, a layer of 1 to 2 mm of bone must be removed at the periphery of the cystic cavity [26].

The patient underwent cyst enucleation, mesiodens impacted tooth odontectomy, periapical exploration of #11 and #12, followed by apical resection of the teeth. Apical #11 and #21 were closed with MTA and GIC and planned orthograde root canal filling for tooth 11 and pulpectomy for #21 after surgery.

In this case, MTA was chosen because preclinical studies clearly showed that MTA has a high sealing capability, good material stability, and excellent biocompatibility. Multiple experimental studies in animals highlighted the mild tissue reactions observed adjacent to this material. Furthermore, histological analysis of the periapical regions demonstrated a frequent deposition of new cementum not only onto the resection plane (cut dentinal surface) but also directly onto MTA. For these reasons, MTA is considered a bioactive material.

In addition, germinectomy for tooth buds #18, #28, #38, and #48 was also performed under general anesthesia. General anesthesia was chosen because based on the CBCT interpretation, the lesion was in contact with the floor of the nasal cavity, and the patient also required the extraction of the four wisdom teeth.

Obturation of #11 was performed one week after surgery and was followed by root canal treatment on #21. In the three- and six-month follow-ups, there were no complaints subjectively. Objectively, there were no signs of swelling, redness, or tooth mobility in #11 and #21. Panoramic and periapical X-rays showed the hermetic filling of #11 and #21, radiopaque area with several radiolucent areas apical to #11, and #12 with an impression of the bone ossification process. There are no signs of recurrence.

The patient was scheduled for periodic follow-up appointments once a year to see if there are any complaints and if signs of healing have formed.

This study is expected to provide insight into how to determine the diagnosis of a lesion through clinical approaches and radiographic and pathological examinations, especially when the lesion involves surrounding structures such as adjacent tooth roots. Cross-sectoral collaboration is needed to be able to handle complex cases to achieve complete care for the patient.

## 5. Conclusion

Our case represents the classical features of the calcifying odontogenic cyst. The comprehensive and multidisciplinary approach is the best way to manage complex cases of mesiodens impaction with COC to relieve symptoms and assure total healing. A proper diagnostic setup is the starting point to develop an efficient treatment plan.

## Data Availability

The data used to support the findings of this study are available from the corresponding author upon request.
